# Meta-Analysis: Diagnostic Accuracy of Antinuclear Antibodies, Smooth Muscle Antibodies and Antibodies to a Soluble Liver Antigen/Liver Pancreas in Autoimmune Hepatitis

**DOI:** 10.1371/journal.pone.0092267

**Published:** 2014-03-20

**Authors:** Wen-Chao Zhang, Feng-Rong Zhao, Juan Chen, Wei-Xian Chen

**Affiliations:** 1 The Department of Laboratory Medicine, the Second Affiliated Hospital of Chongqing Medical University, Chongqing, China; 2 The Department of Gynaecology and Obstetrics, Youyang People’s Hospital, Chongqing, China; 3 Institute of Viral Hepatitis, the Second Affiliated Hospital of Chongqing Medical University, Chongqing, China; Thomas Jefferson University, United States of America

## Abstract

**Background:**

Antinuclear antibodies (ANA), smooth muscle antibodies (SMA) and antibodies to a soluble liver antigen/liver pancreas (anti-SLA/LP) are useful markers that can help clinicians to diagnose and classify autoimmune hepatitis (AIH).

**Objectives:**

To determine whether ANA, SMA and anti-SLA/LP help to accurately diagnose patients with AIH.

**Search strategy:**

The PubMed, CNKI, WANFANG, and SinoMed databases were accessed to retrieve studies published in English and Chinese. Studies published up to October 2013 were reviewed.

**Selection criteria:**

Studies on the diagnostic value of ANA, SMA or anti-SLA/LP in the diagnosis of known or suspected AIH were included.

**Data collection and analysis:**

Two authors evaluated studies independently and rated their methodological quality using quality assessment of diagnostic accuracy studies (QUADAS) tools; relevant data were abstracted. The random-effects method was used to summarize sensitivities, specificities, positive and negative likelihood ratios, and diagnostic odds ratios (DORs) from all 29 studies.

**Results:**

The pooled sensitivity, specificity, positive and negative likelihood ratios, and DOR for ANA were 0.650 (95% confidence interval [CI], 0.619 to 0.680), 0.751 (95%CI, 0.737 to 0.764), 3.030 (95%CI, 2.349 to 3.910), 0.464 (95%CI, 0.356 to 0.604), and 7.380 (95%CI, 4.344 to 12.539), respectively. For SMA, the values were 0.593 (95%CI, 0.564 to 0.621), 0.926 (95%CI, 0.917 to 0.934), 11.740 (95%CI, 7.379 to 18.678), 0.449 (95%CI, 0.367 to 0.549), and 31.553 (95%CI, 17.147 to 58.060), respectively. Finally, for anti-SLA/LP, the values were 0.194 (95%CI, 0.168 to 0.222), 0.989 (95%CI, 0.985 to 0.993), 11.089 (95%CI, 7.601 to 16.177), 0.839 (95%CI, 0.777 to 0.905), and 16.867 (95%CI, 10.956 to 25.967), respectively.

**Authors’ conclusions:**

ANA provided moderate sensitivity and specificity, while SMA gave moderate sensitivity and high specificity, and anti-SLA/LP exhibited low sensitivity and high specificity. All three antibodies were limited by their unsatisfactory sensitivities and lack of consistency.

## Introduction

Autoimmune hepatitis (AIH) was first used as a descriptive term in 1965 [1], although it has been researched extensively, no cure has yet been found. AIH is a chronic progressive and predominantly periportal hepatitis that is characterized by higher prevalence in females than in males, interface hepatitis, hypergammaglobulinemia and autoantibodies [2], [3]. The etiology of AIH is unknown, but both the genetic composition of particular population groups and environmental exposures are involved in its expression. AIH is associated with particular human leucocyte antigens (HLA) alleles, specifically with the ancestral B8-DR3 haplotype and DR4 [4]–[7]. AIH does not exhibit pathognostic symptoms or signs and thus its diagnosis should combine an accurate exclusion of other possible causes of liver disease through a series of clinical, serological, histological and genetic parameters that has been established and revised by a panel of experts [8]–[18]. When diagnosed correctly, AIH is extremely responsive to immunosuppressive therapy [7], [19]. The rapidity and level of this response depends on disease severity, age, and type of presentation [20]. Liver transplantation remains the only therapeutic approach for the end stage of liver disease, and 80 percent of these patients who have undergone a transplant survive after five years.

Based on serological markers, two types of AIH–type 1 (AIH-1) and type 2 (AIH-2) have been classified [20], [21], [34], [35], but they have not yet been established as valid clinical or pathological entities [9]. A proposed third type (AIH-3) has been abandoned, as its serologic marker, antibodies to a soluble liver antigen (anti-SLA), is also found in both other types [22], [74]–[76]. AIH-1 is the most common form of the disease. It affects all ages and is characterized by antinuclear antibodies (ANA) and smooth muscle antibodies (SMA). Anti-SLA have emerged as possible prognostic markers that could help to identify patients with severe AIH, who are prone to relapse after corticosteroid withdrawal [49], [76]–[81]. AIH-2 is marked by the presence of antibodies to liver and kidney microsomes type 1 (anti-LKM-1) [21] and/or liver cytosol antibodies (anti-LC1) and/or antibodies to liver and kidney microsomes type 3 (anti-LKM-3); it is predominantly in infant and juvenile patients.

The lupus erythematosus (LE) cell was first discovered by Hargraves and colleagues [23], and over time it was recognized that the LE cell phenomenon was related to a serum factor reacting with nuclear antigens. This was subsequently termed antinuclear factor (ANF), and later, antinuclear antibodies (ANA). Serum antibodies with specificity for cell nuclear antigens were originally described by Miescher et al. in 1954 [24]. In 1956, a positive test for LE cells in blood was reported in young women suffering from chronic hepatitis, leading to the designation of lupoid hepatitis, an early label for what is now known as AIH-1 [25], [26]. A large number of nuclear molecular targets have been detected, including histones, centromere, chromatin, double-stranded DNA, and ribonucleoprotein complexes, but no single pattern or combination of patterns has been found to be characteristic of AIH [27]. However, none of them are specific for AIH-1, as they have also been identified in rheumatic and infectious diseases. The idea that patients with AIH and systemic lupus erythematosus (SLE) share one or more gene loci that determine ANA reactivity may be demonstrated in future population genome studies. The detection of ANA using indirect immunofluorescence (IIF) was introduced in the early 1960 s [28], and this remains the standard diagnostic screening procedure [29].

Smooth muscle antibodies were initially detected in serum samples of patients with liver diseases by Johnson *et al.* in 1965 [30]. The presence of SMA in patients with autoimmune liver disease was confirmed by Whittingham *et al.* [31]. SMA staining of the arterial vessels (V), glomerular mesangium (G) and fibers surrounding the kidney tubules (T) were reported by Bottazzo et al. [32]. The association between SMA and anti-actin antibodies in AIH was established in 1973 [33]. Anti-SMA of the VGT pattern was confined to be an aggressive form of AIH-1; this is considered specific to AIH-1 [29]. SMA can also be detected using IIF [29], fibroblasts, or HEp-2 cells. Recently, immunometric methods have been developed, such as enzyme-linked immunosorbent assay (ELISA) and immunodot, as well as a new IIF method for detecting antibodies in filamentous actin (F-actin).

Soluble liver antigen (SLA) was first reported as a component of the supernatant of liver and kidney homogenates by Manns and colleagues in 1987 [34]. Berg’s group [35] found that the liver pancreas (LP) antigen was also present in the supernatant of liver and pancreas homogenates. The anti-SLA and anti-LP have been shown to target the same antigen, hence the current term, anti-SLA/LP antibodies [34]–[36]. Anti-SLA antibodies have also been proposed as markers of a third type of severe AIH that is seronegative for the conventional AIH-1 auto-antibodies [35]. Beyond the conventional competitive inhibition ELISA originally used for anti-SLA antibody detection, the identification of the molecular target of anti-SLA/LP antibodies as the UGA serine tRNA-associated protein has led to the development of new ELISA kit or dot-blot assays [36], [37].

Although ANA, SMA and anti-SLA/LP are now used by clinicians to help diagnose AIH, their performances in practice have not been assessed systematically. In this report, we summarize published data on the sensitivity, specificity, positive and negative likelihood ratios and diagnostic odds ratios (DORs) of ANA, SMA and anti-SLA/LP for diagnosing AIH. We then assessed their diagnostic accuracies in clinical practice.

## Methods

### Data Sources and Study Selection

We developed a review protocol and followed standard reporting guidelines [38]. The PubMed, CNKI, WANFANG, and SinoMed databases were searched for studies that examined ANA, SMA or anti-SLA/LP association with AIH and were published up to October 2013, in English and Chinese. Our searches were based on combinations of the following index terms: *autoimmune hepatitis, AIH, and antinuclear antibody(ies), (anti-) smooth muscle antibody(ies), antibody(ies) to a soluble liver antigen/liver pancreas, and nuclear antigen(s), smooth muscle antigen(s), soluble liver antigen(s)/liver pancreas, and ANA, SMA, SLA/LP, anti-SMA, anti-SLA/LP antibody(ies).* We also reviewed the reference lists of retrieved studies and reviewed articles.

Two reviewers independently scanned abstracts that met the inclusion criteria. We included studies that evaluated the utility of assaying ANA, SMA or anti-SLA/LP for diagnosis of confirmed or suspected AIH, and that provided sufficient data to allow calculation of sensitivity and specificity in diagnosis. We used the standards of the International Autoimmune Hepatitis Group (IAIHG) [8], [9] and the guideline approved by the American Association for the Study of Liver Diseases (AASLD) [16], [17] as reference standards for AIH. The following articles were not included in the current study: reviews; publications without valid data to obtain the sensitivity and specificity of ANA, SMA, and anti-SLA/LP; researches not related to the diagnostic values of ANA, SMA, and anti-SLA/LP for AIH.

### Data Extraction and Study Quality Assessment and Data Analysis

We extracted data using a standard form that included the author, publication year, demographic characteristics of the participants, methods of antibody testing, true positive results, false negative results, true negative results, false positive results, sensitivity, and specificity. Two investigators independently assessed the methodological quality of each study using 14 standard items from the quality assessment of diagnostic accuracy studies (QUADAS) tool, which is a quality assessment tool specifically developed for systematic reviews of diagnostic accuracy studies [39]. We resolved any item discrepancies through discussion.

We used the random-effects model to combine estimates of sensitivity, specificity, positive and negative likelihood ratios, and DORs [40], [41]. We conducted threshold analyses and meta-regression to assess whether heterogeneity and a threshold effect existed among the examined studies [42], [43]. We investigated heterogeneity using stratified analyses for different assays and AIH patients’ race.

We examined funnel plots for DORs to explore the possibility of publication bias [44]. We used MetaDiSc Version 1.4 and Review Manager Version 5.2 software for our analyses.

## Results

### Search Results and Characteristics of Studies and Study Quality

**Figure 1:**
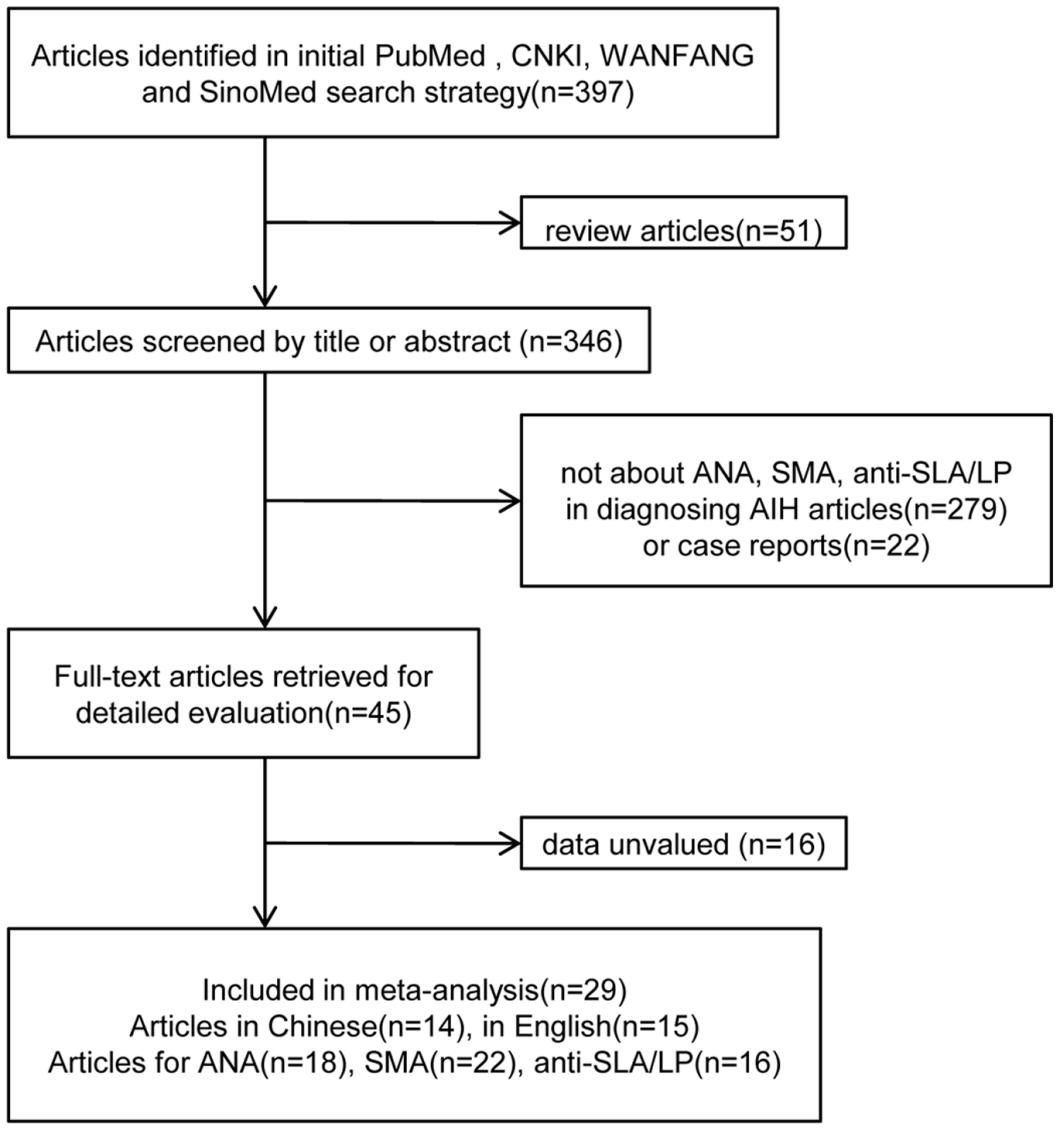
Study flow diagram.

We identified 397 articles, of which 29 met the inclusion criteria (Figure 1) [45]–[73]. Of these, 15 (51.7%) studies were in English [45]–[59] and 14 (48.3%) were in Chinese [60]–[73]. Eighteen studies on 968 patients investigated the diagnostic accuracy of ANA [45]–[47], [50], [52], [54], [59]–[62], [64]–[68], [70], [71], [73], 22 studies on 1,193 patients reported on the diagnostic accuracy of SMA [46], [47], [50], [52]–[55], [57]–[59], [60]–[65], [67]–[71], [73], and 16 studies on 850 patients focused on the diagnostic accuracy of anti-SLA/LP [46]–[52], [56], [60], [63], [65], [68]–[70], [72], [73].

Table 1 summarizes the characteristics of all included studies [45]–[73]. In the ANA, SMA and anti-SLA/LP studies, the mean ages of patients were 42.43 years, 43.47 years and 36.90 years, respectively, and the percentage of females were 74.62%, 74.35% and 68.31%, respectively. The prevalence of ANA, SMA and anti-SLA/LP in AIH patients was examined in different geographical regions, including China, Japan, Iraq, Germany, France, Italy, Turkey, the United Kingdom, Austria and Sweden. Most studies of ANA and SMA employed IIF. In total, 75% (12 of 16) of anti-SLA/LP studies used immunoblot assays, 18.75% (3 of 16) used ELISA, and only 6.25% (1 of 16) used radioligand assay (RLA). The cutoff values for positive test results reported in the studies were inconsistent.

**Table 1:** Characteristics of studies of ANA, SMA and SLA/LP.

Study (No.)	Patients race	Setting	Assay (cut-off)	AIH patients	Women (%)	Mean^1^, Median^2^ age, (SD) years	Age range, years	Control participants	design	Golden standard (No.)
45	Japanese	not referred	IFT(1∶160*)	16	14(87.50)	59.00^2^	25–69	AIC(n = 21), PBC(n = 37), CVH(n = 21)	retrospective	8
46	German	not referred	IFT(1∶80*#), ELISA(&)	121	29(23.97)	40.99^1^	not referred	PBC(n = 107), SLE(n = 17), MCTD(n = 16), Arthritis(n = 9), HC(n = 100)	retrospective	unclear
47	Iraqi	Teaching Hospital	IFT(1∶20*, 1∶40# ), Immunoblot(&)	50	45(90.00)	27.20^1^(9.44)	8–62	CVH(n = 50), HC(n = 30)	retrospective	unclear
48	Chinese	Peking Union Medical College Hospital	ELISA(20RU/mL, &)	44	10(22.72)	47.80^1^(11.50)	8–82	PBC(n = 198), LDC(n = 41)	retrospective	9
49	English	King’s College Hospital	RLA(&)	66	48(72.73)	13.00§ 8.00¶	3–20	ASC(n = 17), PBC(n = 20), CVH(n = 40), LDC(n = 41), SLE(n = 16), CD(n = 8), Diabetes(n = 10), Polymyositis(n = 12), HC(n = 56)	retrospective	9
50	Japanese	not referred	IFT(1∶40*#), Immunoblot(&)	80	69(86.25)	54.56^1^	not referred	PBC(n = 46), PSC(n = 10), CVH(n = 47), SLE(n = 48), Cryptogenic hepatitis(n = 3), HC(n = 40)	retrospective	9
51	Japanese	Kagawa Medical University	ELISA(&)	18	not referred	not referred	not referred	CVH(n = 150), NBNC(n = 20), HC(n = 30)	retrospective	8, 10
52	Italian and Turkish	not referred	IFT(1∶40*#), Immunoblot(&)	192	157(81.77)	43.11^1^	3–82	AIH/PBC(n = 30), PBC(n = 170), AIH/PSC(n = 9), PSC(n = 45), AIH/CVH(n = 11), CVH(n = 292), NASH(n = 135), DILI(n = 86), Wilson’s disease(n = 16)	retrospective	11
53	German	University Medical Centre Hamburg-Eppendorf	IFT(1∶40#)	35	26(74.29)	49.00^1^	21–68	CVH(n = 34), PBC(n = 21), PSC(n = 5), NASH(n = 14), LDC(n = 13), Wilson’s disease(n = 1)	prospective	unclear
54	Italian	Semeiotica Medica II and Clinica Medica II	IFT(1∶40*#)	35	not referred	not referred	not referred	CVH(n = 290)	retrospective, consecutive	8
55	Italian	not referred	IFT(1∶80#)	55	not referred	not referred	not referred	PBC(n = 20), CVH(n = 20), HC(n = 25)	retrospective, consecutive	9
56	Austrian	not referred	Immunoblot(&)	51	not referred	40.00^1^	7–63	CVH(n = 10), HC(n = 10)	retrospective	8, 12, 13, 14
57	Italian	not referred	IFT(1∶40#)	41	not referred	53.20^2^	18–83	PBC(n = 40), CVH(n = 30), CD(n = 16), Steatosis(n = 10)	retrospective	9
58	Italian	S. Orsola-Malpighi Hospital	IFT(1∶40#)	100	not referred	39.00^2^	7–82	PBC(n = 20), CVH(n = 51), CD(n = 17), HC(n = 50)	retrospective, consecutive	9
59	Swedish	Linköping University Hospital	IFT(1∶80*#)	46	34(73.91)	47.00^1^	21–28	NAFLD(n = 50), UC(n = 53), PSC(n = 27), Crohn’s disease(n = 51), HC(n = 40)	retrospective	9, 15
60	Chinese	not Teaching Hospital	IFT(*#), Immunoblot(&)	45	not referred	not referred	not referred	SLE(n = 20), HC(n = 20)	retrospective	unclear
61	Chinese	Second Hospital Affiliated to NanChang University	IFT(1∶100*#)	68	57(83.82)	44.00^1^	4–62	PBC(n = 41), PSC(n = 52), CVH(n = 276), HC(n = 50)	retrospective	16
62	Chinese	Second Hospital Affiliated to NanChang University	IFT(*#)	56	49(87.5)	44.00^1^	4–67	PBC(n = 32), CVH(n = 134), HC(n = 40)	retrospective	17
63	Chinese	not referred	IFT(#), Immunoblot(&)	11	not referred	not referred	not referred	PBC(n = 3), PSC(n = 11), CVH(n = 25),	retrospective	9
64	Chinese	not Teaching Hospital	IFT(*#)	32	21(65.63)	49.70^1^(18.30)	not referred	CVH(n = 60)	retrospective	17
65	Chinese	not referred	IFT(*#), Immunoblot(&)	22	17(77.27)	35.00^1^ (5.00)	17–51	PBC(n = 43), PSC(n = 3)	retrospective	unclear
66	Japanese	not referred	IFT(*)	8	not referred	not referred	not referred	PBC(n = 57), CVH(n = 363), CTD(n = 151)	retrospective	unclear
67	Chinese	Second Hospital Affiliated to NanChang University	IFT(*#)	63	54(85.71)	43.00^1^	4–69	PBC(n = 36), PSC(n = 25), CVH(n = 145), HC(n = 50)	retrospective	18
68	Chinese	not Teaching Hospital	IFT(*#), Immunoblot(&)	31	23(74.19)	40.56^1^(8.23)	24–63	CVH(n = 32), HC(n = 31)	retrospective	unclear
69	Chinese	not Teaching Hospital	IFT(#), Immunoblot(&)	7	5(71.43)	not referred	36–60	PBC(n = 11), LDC(n = 20)	retrospective	9
70	Chinese	Second Hospital Affiliated to NanChang University	IFT(*#), Immunoblot(&)	31	28(90.32)	42.00^1^	4–67	PBC(n = 29), CVH(n = 46), HC(n = 40)	retrospective	17
71	Chinese	not referred	IFT(1∶100*#)	25	not referred	not referred	36–67	PBC(n = 19), PSC(n = 2), HC(n = 60)	retrospective	8
72	Chinese	Beijing You’an Hospital, Affiliated to Capital Medical University	Immunoblot(&)	34	not referred	53.00^1^(12.00)	not referred	AIH/PBC(n = 6), PBC(n = 20), CVH(n = 29), HC(n = 20)	retrospective	16
73	Chinese	Second Hospital Affiliated to NanChang University	IFT(*#), Immunoblot(&)	47	41(87.23)	44.00^1^	4–67	PBC(n = 32), CVH(n = 116), HC(n = 40)	retrospective	17

*/#/&: cut-off and assays for ANA/SMA/SLA. ^1^/^2^: mean/median age of AIH patients. §/¶: median age of AIH-1/2.

IIF = indirect immunofluorescence, ELISA = enzyme-linked immunosorbent assay, RLA = radioligand assay. ANA = antinuclear antibodies, SMA = anti smooth muscle antibodies, SLA/LP = antibodies to a soluble liver antigen/liver pancreas, AIH = autoimmune hepatitis, PBC = primary biliary cirrhosis, AIC = autoimmune cholangitis/cholangiopathy, CVH = chronic viral hepatitis B or C, SLE = systemic lupus erythematosus, MCTD = mixed connective tissue disease, HC = healthy controls, LDC = liver disease controls, CD = coeliac disease, PSC = primary sclerosing cholangitis, NBNC = chronic hepatitis-non-B, non-C, DILI = drug-induced liver injury, NASH = non-alcoholic steatohepatitis, UC = ulcerative colitis, NAFLD = non-alcoholic fatty liver disease, CTD = collagen diseases.

The characteristics of the control groups varied among ANA, SMA and anti-SLA/LP studies; the groups included healthy individuals and patients with other non-AIH diseases like autoimmune liver diseases (e.g., primary biliary cirrhosis [PBC], primary sclerosing cholangitis [PSC], autoimmune cholangitis/cholangiopathy), rheumatic diseases (e.g., SLE, mixed connective tissue disease, collagen diseases, polymyositis), and other liver diseases (e.g., chronic viral hepatitis, cryptogenic hepatitis, drug-induced liver injury, non-alcoholic steatohepatitis, Wilson’s disease, non-alcoholic fatty liver disease). Patient enrollment was retrospective in all ANA and anti-SLA/LP studies, as well as 17 of the 18 SMA studies. We observed that the scoring systems for diagnosing AIH varied among the included studies.

**Figure 2:**
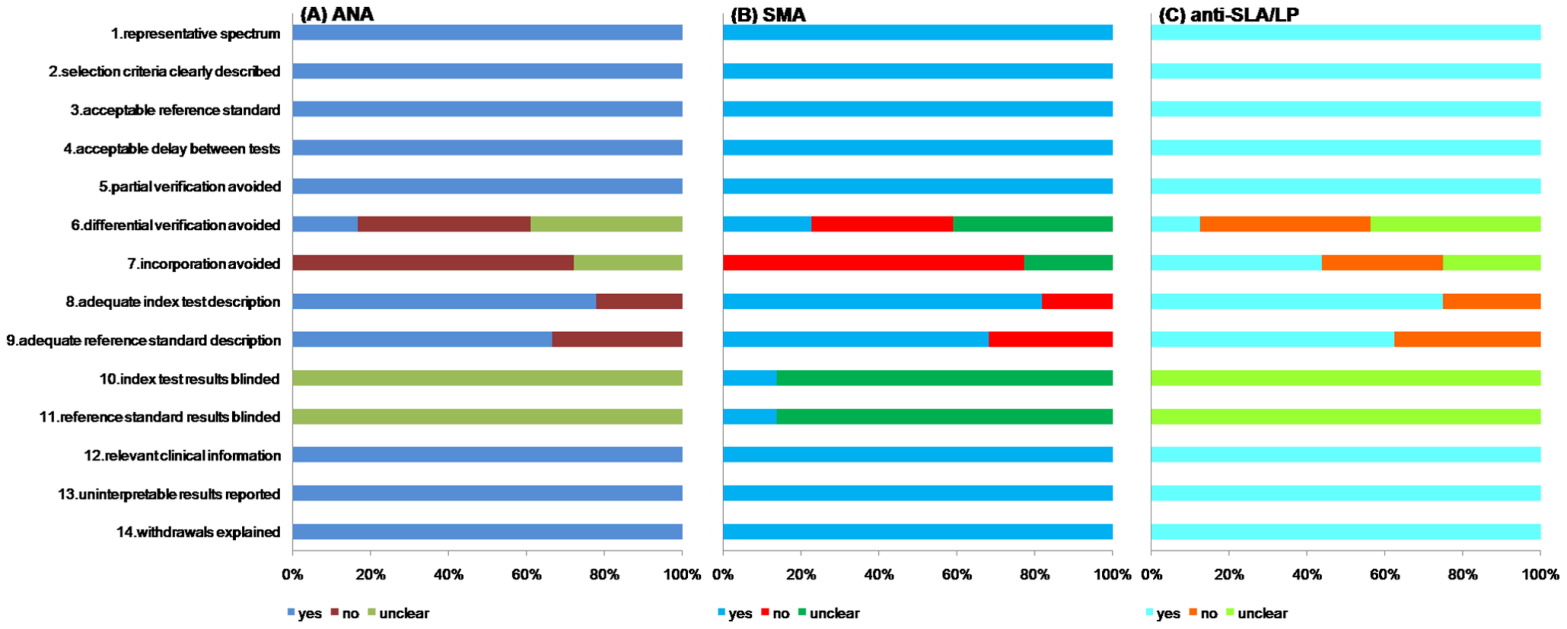
A cumulative bar plot of methodological quality items across ANA, SMA and anti-SLA/LP studies.

All studies that were included in the meta-analysis were checked against each item tested with the QUADAS tool and rated as “yes”, as “no” if they did not, and as “unclear” if there was insufficient data on the subject in the study. They had high quality and satisfied at least eight out the 14 items. The median score for quality among the ANA, SMA and anti-SLA/LP studies was consistently 11. None of the studies satisfied all the criteria on the quality checklist. Item 10 and 11, index test results blinded and reference standard blinded to index test, were “unclear” for all studies except for three [53], [57], [58]; all of these were SMA studies. Eight articles did not fulfill item 9, which was the use of adequate referencing standards [46], [47], [53], [60], [63], [65], [66], [68]. The tests for ANA, SMA and anti-SLA/LP in five articles were not described in detail [60], [65], [66], [68], [70], so they did not fulfill item 8 (adequate index test description). As the two auto-antibodies (ANA and SMA) that were accepted as diagnostic markers included in numerous criteria designed to establish AIH, some differences surfaced among ANA, SMA and anti-SLA/LP studies concerning item 7 (incorporation avoid). Three articles only included anti-SLA/LP studies and were classified as “yes” [48], [49], [56], whereas one article [72] included in anti-SLA/LP studies received a “no” as its reference standard [16]. Furthermore, 15 articles belonging to ANA or SMA studies were “no” [45], [46], [52], [54]–[59], [61], [62], [64], [67], [70], [71], and six articles were “unclear”, as they lacked well-defined reference standards [47], [53], [60], [65], [66], [68]. Another four articles would satisfy item 7 only these were anti-SLA/LP studies [50], [63], [69], [73]. There were only five articles [55], [58], [62], [70], [73] that conformed to item 6 (differential verification avoided) (see Figure 2).

### Diagnostic Accuracy of ANA, SMA and Anti-SLA/LP

**Figure 3:**
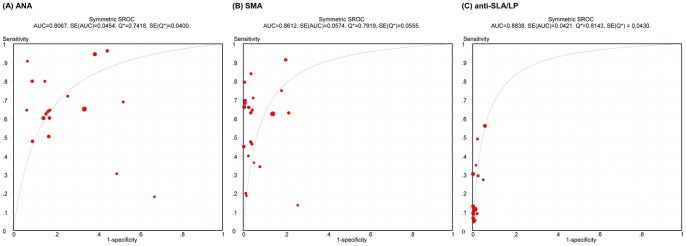
SROC curves for ANA, SMA and anti-SLA/LP. Each solid circle represents each study in the meta-analysis. The size of each study is indicated by the size of the solid circle. SROC = summary receiver operative curve, ANA = antinuclear antibodies, SMA = smooth muscle antibodies, anti-SLA/LP = antibodies to a soluble liver antigen/liver pancreas, AUC = area under the curve, SE = standard error, Q* = Cochran Q.

For ANA, the overall positive and negative likelihood ratios were 3.030 (95% confidence interval [CI], 2.349 to 3.910) and 0.464 (95%CI, 0.356 to 0.604), respectively. The pooled sensitivity was 0.650 (95%CI, 0.619 to 0.680) and the specificity was 0.751 (95%CI, 0.737 to 0.764). The overall DOR was 7.380 (95%CI, 4.344 to 12.539; Table 2). Data that were calculated from ANA provided moderate diagnostic value for AIH, but significant heterogeneity was found among included studies. The summary receiver operative curve (SROC) did not show a clear ROC type of trade-off between sensitivity and specificity (Figure 3A), at the same time, the Spearman correlation coefficient was −0.085 (p = 0.738). This suggests that there were no remarkable threshold effects in these ANA studies. Therefore, we next examined the reasons for heterogeneity through a meta-regression analysis and discovered that heterogeneity was caused by the different races of patients; thus, we performed a stratified meta-analysis for each subgroup.

**Table 2:** Summary values of ANA, SMA and anti-SLA/LP for all studies and different subgroups.

	Groups (No.)	Sensitivity	Specificity	LR+	LR-	DOR	Spearman correlation coefficient
	**ANA**						
Summary accuracy (95% CI)(¢)	1(n = 18)	0.650 (0.619–0.680)	0.751 (0.737–0.764)	3.030 (2.349–3.910)	0.464 (0.356–0.604)	7.380 (4.344–12.539)	−0.085
	1$(n = 4)	0.599 (0.549–0.648)	0.770 (0.749–0.791)	3.980 (1.962–8.074)	0.515 (0.407–0.653)	8.306 (3.638–18.968)	−0.200
	1&(n = 14)	0.685 (0.646–0.723)	0.738 (0.720–0.756)	2.793 (2.079–3.751)	0.438 (0.293–0.655)	7.117 (3.452–14.674)	−0.110
heterogeneity P value(£)	1(n = 18)	0.000	0.000	0.000	0.000	0.000	0.738
	1$(n = 4)	0.001	0.000	0.000	0.027	0.000	0.800
	1&(n = 14)	0.000	0.000	0.000	0.000	0.000	0.708
	**SMA**						
Summary accuracy (95% CI)(¢)	2(n = 22)	0.593 (0.564–0.621)	0.926 (0.917–0.934)	11.740 (7.379–18.678)	0.449 (0.367–0.549)	31.553 (17.147–58.060)	−0.051
	2$(n = 8)	0.596 (0.556–0.634)	0.884 (0.869–0.898)	6.584 (4.059–10.681)	0.443 (0.349–0.563)	20.703 (9.740–44.003)	0.214
	2&(n = 14)	0.590 (0.548–0.631)	0.969 (0.960–0.977)	15.462 (7.338–32.578)	0.458 (0.334–0.627)	37.159 (15.007–92.011)	−0.046
heterogeneity P value(£)	2(n = 22)	0.000	0.000	0.000	0.000	0.000	0.820
	2$(n = 8)	0.000	0.000	0.000	0.000	0.000	0.610
	2&(n = 14)	0.000	0.000	0.000	0.000	0.000	0.876
	**anti-SLA/LP**						
Summary accuracy (95% CI)(¢)	3(n = 16)	0.194 (0.168–0.222)	0.989 (0.985–0.993)	11.089 (7.601–16.177)	0.839 (0.777–0.905)	16.867 (10.956–25.967)	0.450
	3$(n = 4)	0.281 (0.239–0.325)	0.983 (0.974–0.989)	12.934 (5.883–28.432)	0.638 (0.467–0.871)	20.850 (9.163–47.443)	0.800
	3&(n = 12)	0.103 (0.075–0.136)	0.995 (0.990–0.998)	11.334 (5.658–22.706)	0.919 (0.885–0.954)	14.093 (6.742–29.459)	0.427
	3‡(n = 12)	0.139 (0.113–0.170)	0.992 (0.987–0.996)	10.955 (6.437–18.646)	0.892 (0.844–0.941)	13.262 (7.572–23.228)	0.664
	3†(n = 4)	0.323 (0.266–0.384)	0.984 (0.973–0.991)	22.955 (4.524–116.465)	0.734 (0.554–0.973)	30.765 (9.268–102.13)	0.400
heterogeneity P value(£)	3(n = 16)	0.000	0.000	0.839	0.000	0.872	0.080
	3$(n = 4)	0.000	0.000	0.141	0.000	0.176	0.200
	3&(n = 12)	0.020	0.043	0.952	0.165	0.966	0.167
	3‡(n = 12)	0.000	0.060	0.985	0.000	0.983	0.018
	3†(n = 4)	0.000	0.000	0.066	0.000	0.231	0.600

¢: random-effects model; £: random-effects heterogeneity. 1/2/3: three groups (ANA/SMA/anti-SLA/LP), $/&: subgroups for European/Asian patients of autoimmune hepatitis, ‡/†: subgroups for immunoblot assay/non-immunoblot assay of detecting anti-SLA/LP.

ANA = antinuclear antibodies, SMA = smooth muscle antibodies, anti-SLA/LP = antibodies to a soluble liver antigen/liver pancreas, No. = the number of included articles, LR+/− = positive/negative likelihood ratio, DOR = diagnostic odds ratio, CI = confidence interval.

**Figure 4:**
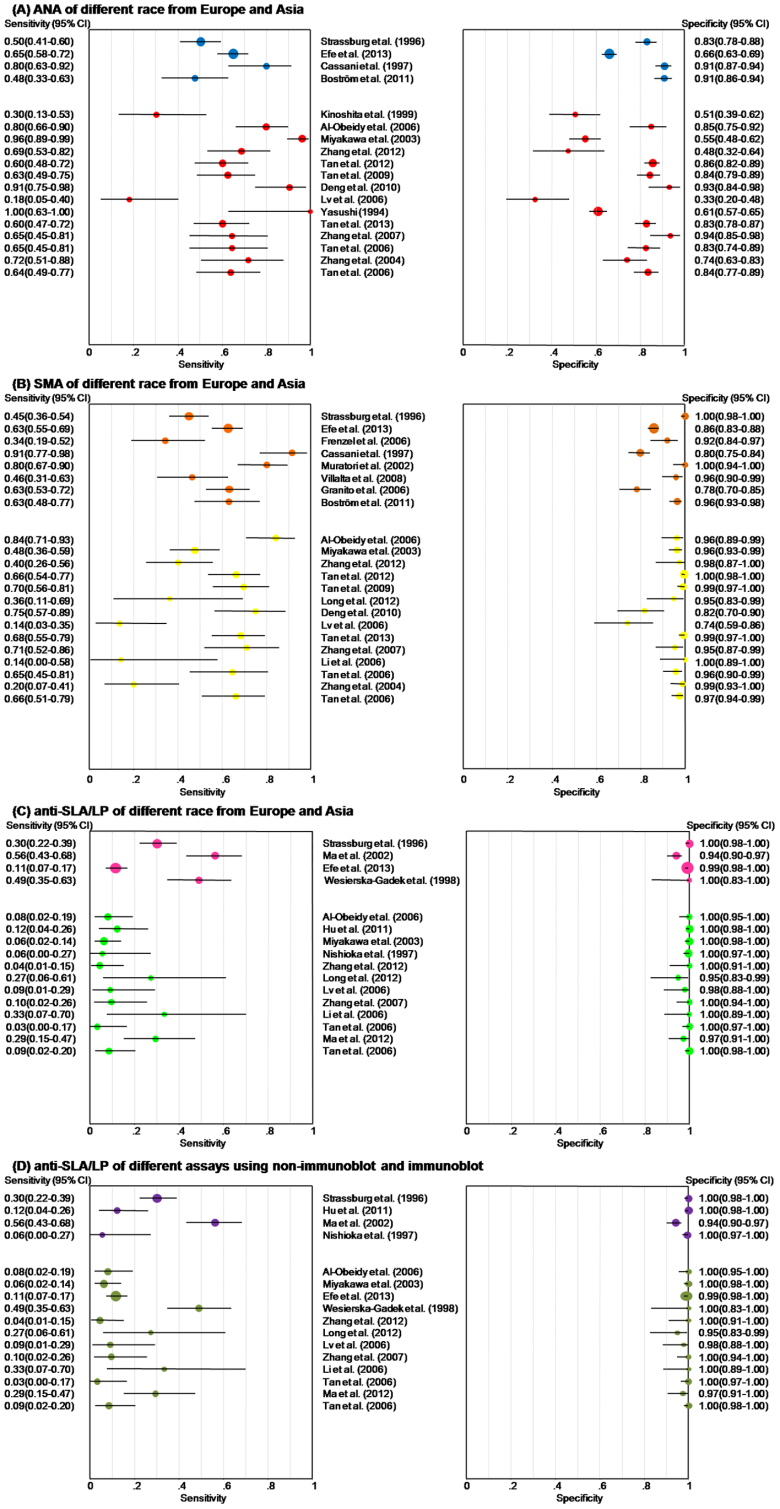
Forest plots of estimates of sensitivity and specificity for different subgroups. Each solid circle indicates the point estimate of sensitivity and specificity from each study in the meta-analysis. Error bars indicate 95% confidence intervals. There are different colors presenting subgroups. As for different race, blue, orange and pink represent Europe, and red, yellow and green represent Asia in three groups (ANA, SMA and anti-SLA/LP), respectively. Meanwhile, olive is immunoblot and purple is non-immunoblot assay for different assays of detecting anti-SLA/LP. ANA = antinuclear antibodies, SMA = smooth muscle antibodies, anti-SLA/LP = antibodies to a soluble liver antigen/liver pancreas.

The solid blue circles in Figure 4A show the forest plots for sensitivity and specificity estimates from the four studies that used AIH patients from Europe. This subgroup still showed moderate sensitivity and specificity, with pooled sensitivity and specificity of 0.599 (95%CI, 0.549 to 0.648) and 0.770 (95%CI, 0.749 to 0.791), respectively. Table 2 shows the results of the meta-analysis. The overall values from the four studies were almost the same in terms of heterogeneity as those from the meta-analysis of all 18 studies. Furthermore, the threshold effects were negative, as shown by the Spearman correlation coefficient of −0.2 (p = 0.8).

The solid red circles in Figure 4A show the forest plots for sensitivity and specificity estimates from the 14 studies that used AIH patients from Asia. Similar to the results from the four studies with European AIH patients, moderate sensitivity estimates (0.685, 95%CI, 0.646 to 0.723) and specificity estimates (0.738, 95%CI, 0.720 to 0.756) were demonstrated. However, no statistically significant threshold effects were found, as the Spearman correlation coefficient was −0.11 (p = 0.708; Table 2).

Our results show that SMA had an extraordinary diagnostic accuracy for AIH with an outstanding specificity (0.926, 95%CI, 0.917 to 0.934), positive likelihood ratios (11.740, 95%CI, 7.379 to 18.678), and DOR (31.553, 95%CI, 17.147 to 58.060); however, the results for sensitivity (0.593, 95%CI, 0.564 to 0.621) and the negative likelihood ratios (0.449, 95%CI, 0.367 to 0.549) were moderate. Data from SMA studies did not show evident threshold effects due to an indefinite ROC type of trade-off between sensitivity and specificity (Figure 3B). Moreover, while the Spearman correlation coefficient was −0.051 (p = 0.82), heterogeneity was still apparent. We explored the sources of heterogeneity through meta-regression analysis, and then carried out subgroup analyses for different races.

The solid orange and yellow solid circles in Figure 4B represent the forest plots for sensitivity and specificity estimates from the 8 studies that used AIH patients from Europe and 14 studies with AIH patients from Asia. There was no significant change when the pooled sensitivity, negative likelihood ratios and threshold effects of two subgroups were compared to those from all 22 studies. In terms of pooled specificity, positive likelihood ratios and DOR, the results from all studies were superior to those limited to patients with AIH from Europe and inferior to those limited to AIH patients from Asia.

The pooled sensitivity, specificity, positive and negative likelihood ratios and DOR for anti-SLA/LP were 0.194 (95%CI, 0.168 to 0.222), 0.989 (95%CI, 0.985 to 0.993), 11.089 (95%CI, 7.601 to 16.177), 0.839 (95%CI, 0.777 to 0.905) and 16.867 (95%CI, 10.956 to 25.967), respectively (see Table 2). Threshold effects were indicated by a ROC-type trade-off between sensitivity and specificity (see Figure 3C); the Spearman correlation coefficient (0.45; p = 0.08) was not noteworthy, and non-threshold effects were negative due to the positive likelihood ratio (chi-squared = 9.68, p = 0.839) and DOR (chi-squared = 9.1, p = 0.872). (The results of chi-squared are not shown in Table 2).

Despite the results given above, heterogeneity was still evident in the anti-SLA/LP studies. In order to investigate this, we defined two subgroups based on the different races of AIH patients, and then conducted an analysis with various assays like the immunoblot and non-immunoblot assay.

The solid pink and green circles in Figure 4C show the forest plots for sensitivity and specificity estimates of 4 studies that used AIH patients from Europe and 12 studies that included patients from Asia. Compared with the data from all 16 studies, the summary values in studies of European AIH patients showed lower specificity, higher sensitivity, and similar positive and negative likelihood ratios and DOR. Moreover, the sensitivity, negative likelihood ratios and DOR in studies of AIH patients from Asia were smaller than in all 16 studies taken together, while they showed higher specificity and positive likelihood ratios. Furthermore, no positive threshold effects were found, but heterogeneity was present.

The solid olive and purple circles in Figure 4D show the forest plots for sensitivity and specificity estimates from 12 studies that used immunoblot and 4 studies that used a non-immunoblot assay. Stratified analyses for anti-SLA/LP showed no major differences for the pooled sensitivity, specificity and negative likelihood ratios for the different measurement methods. What surprised us was that positive likelihood ratios and the DOR from the stratified non-immunoblot group were highest in all subgroups and showed no obvious heterogeneity; however, it was also found that the positive threshold effects in the stratified immunoblot group were introduced via the presence of different ethnic populations.

To summarize, we found that ANA demonstrated the highest sensitivity, SMA the highest DORs, and anti-SLA/LP the highest specificity, but were limited by their unfavorable sensitivities and high heterogeneity. When comparing results for ANA, SMA and anti-SLA/LP within the same race, we found that the results for studies of AIH patients from Asia were qualitatively similar to the results for all studies; specifically, Asian patients shared the highest sensitivity estimates for ANA, the best DORs for SMA, and the best specificity estimates for anti-SLA/LP. Studies of AIH patients from Europe differed slightly in that the best DORs appeared in anti-SLA/LP rather than SMA.

### Publication Bias

**Figure 5:**
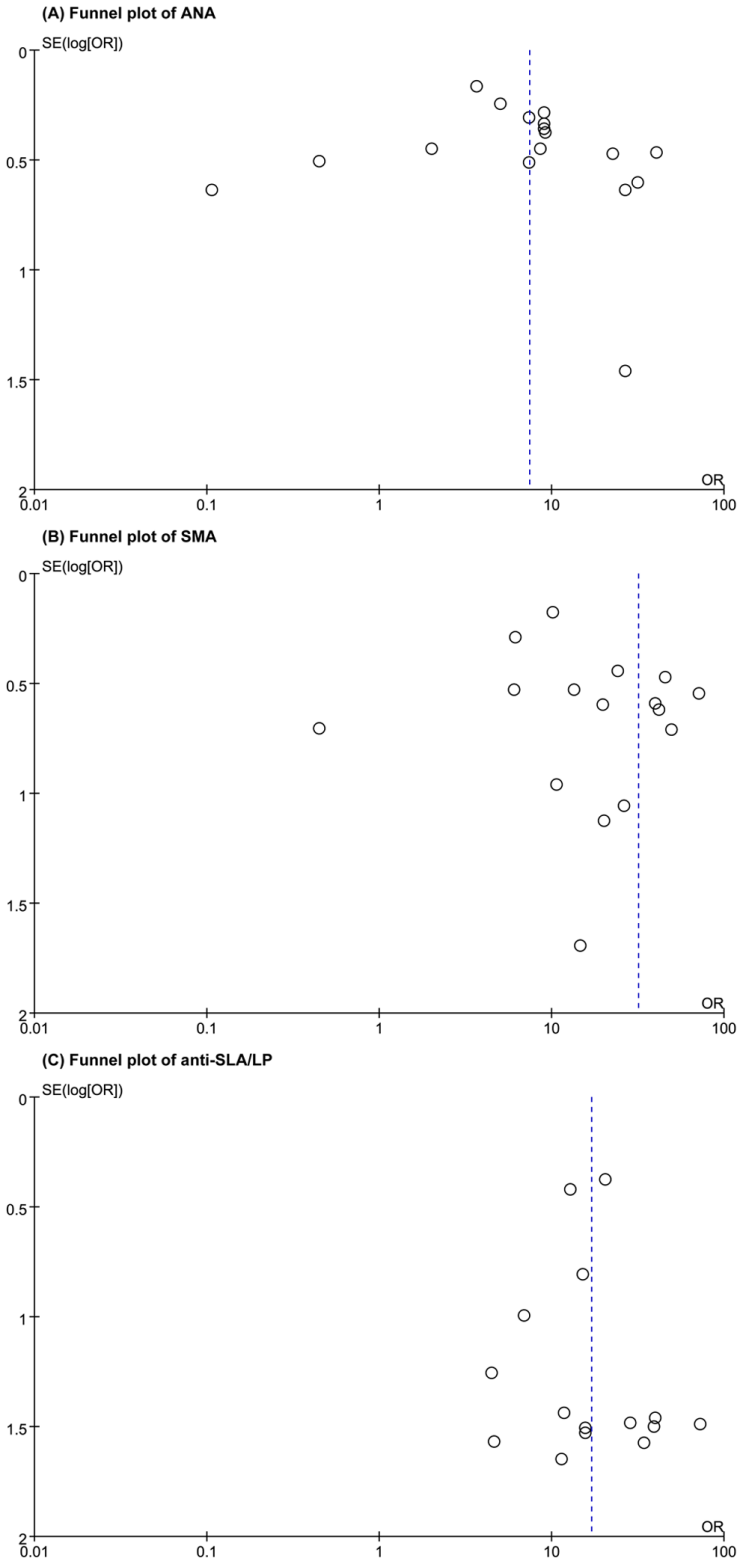
Funnel plots for the assessment of potential publication bias in ANA, SMA and anti-SLA/LP. The funnel graph plots the log of the DOR against the standard error of the log of the DOR (an indicator of sample size). Each open circle represents each study in the meta-analysis. The line in the center indicates the summary DOR. ANA = antinuclear antibodies, SMA = smooth muscle antibodies, anti-SLA/LP = antibodies to a soluble liver antigen/liver pancreas. DOR = diagnostic odds ratio.

Funnel plots for ANA, SMA and anti-SLA/LP were created to test for publication bias, and they showed a degree of asymmetry. This indicates that publication bias was potentially present (Figure 5).

## Discussion

Autoimmune hepatitis is a typically severe inflammation of the liver of unknown cause. It is hypothesized that its pathogenesis comprises environmental triggers, a failure of immune tolerance mechanisms, and a genetic predisposition toward the induction a T cell–mediated immune attack when liver antigens are detected, leading to a progressive necroinflammatory and fibrotic process in the liver [82]. Diagnosing AIH accurately is difficult, as its onset is frequently accompanied by non-specific symptoms like fatigue, jaundice, nausea, abdominal pain, and arthralgias [83]. However, the clinical spectrum we found in the examined studies ranged from widely an asymptomatic presentation [84], [85] to acute and severe diseases [86], [87].

All ages [89], [90] and ethnic groups [90]–[94] are susceptible to AIH, but women are affected more frequently than men, with a gender ratio being 3.6 to 1 [88]. It has been found that prednisone, either alone or in combination with azathioprine, is effective in improving symptoms; however, according to laboratory tests, histologic findings, and survival rates [95]–[97], patients with inactive or burned out cirrhosis cannot benefit from this form of therapy that consists of prednisone and azathioprine [114]. These patients also have an increased risk of drug-induced side-effects due to their associated hypoalbuminemia, hyperbilirubinemia, and portosystemic shunting, which can all affect protein-binding and the disposition of free prednisolone [115].

The diagnosis of AIH is based on a scoring system that was codified by an international panel in 1993 [8] and revised in 1999 [9]. It consists of histologic abnormalities, characteristic clinical and laboratory findings, abnormal levels of serum globulins, and the presence of one or more characteristic autoantibodies. However, PSC and PBC can result in clinical, histological, genetic, and laboratory findings that resemble those of AIH [98]–[104]. Moreover, AIH can have features that resemble cholestatic syndromes [105]–[109]. These non-specific shared features can confound the codified diagnostic scoring system. Consequently, a guideline designed for a more comprehensive diagnosis of AIH was approved by the AASLD [16], [17].

ANA, SMA, anti-LKM-1 and anti-LC-1 constitute the conventional serological repertoire for the diagnosis of AIH, and anti-SLA and atypical perinuclear antineutrophil cytoplasmic antibodies (p-ANCA) [34], [35], [49], [77]–[79] are alternate autoantibodies used for classifying patients into the different AIH types. If clinicians hope to maximize diagnostic efficiency of testing by combining measuring of the above markers, they need to consider the risks and benefits of this approach. It is harmful and costly to treat persons that have false-positive results for AIH; thus, clinical trials and cost-effectiveness studies of trade-offs between measuring all markers and targeted measuring of specific markers are needed.

Our review shows that the diagnostic accuracies of SMA and anti-SLA/LP for AIH were extraordinarily high, as the DOR was 31.553 for SMA and 16.867 for anti-SLA/LP. With a DOR value of 7.380, ANA had a slightly inferior diagnostic accuracy, which was mainly due to the excessive heterogeneity in ANA. Furthermore, the results of our review confirm that using these autoantibodies in the diagnosis of AIH has drawbacks, since they are also present in other liver diseases such as PBC, PSC, drug-induced liver injury, non-alcoholic fatty liver disease, chronic viral hepatitis B or C [45]–[73]. It is reported that various characteristics of autoantibodies testing play important roles in diagnostic sensitivity and specificity. The nuclear targets of ANA in AIH are uncertain, and many ANA in AIH are non-reactive to the major recombinant nuclear antigens. Therefore, clinicians prefer to assess ANA by IIF on Hep-2 cell lines [110] or by an enzyme immunoassay using microtiter plates with adsorbed recombinant or highly purified antigens [111]. In clinical laboratories, SMA is typically confirmed through IIF on murine stomachs and kidneys [27].

Anti-actin has greater specificity for AIH than SMA [112]. A thermolabile F-actin depolymerizing factor has been described in serum, but the best assay for detecting anti-actin has not yet been established [113]. A standardized enzyme immunoassay for anti-SLA/LP has been validated using Western blot for recombinant antigens; a commercial assay is available in Europe [37]. Meanwhile, the quality of studies included in our review also influences sensitivity and specificity of the results. We tested for inconsistency through meta-regression and with Spearman correlation coefficients. Furthermore, we conducted a stratified analysis step for negative threshold effects, but there were still distinct non-threshold effects within each race.

Although we minimized bias as much as we could in our full-scale search strategy, high levels of bias were present in articles and data extraction, meaning that there were indeed limitations to the chosen approach. First, only 29 reports were included, leading to results bias. Second, we could only integrate the available published results and might miss some important ongoing/unpublished research data and the language capability could only allow us to choose the publications in English and Chinese. All of these reasons might produce publication selection bias, and at the same time, our funnel plots suggest that there was a publication bias for favorable ANA, SMA and anti-SLA/LP studies. Third, as ANA, SMA and anti-SLA/LP are incorporated into the current diagnostic criteria of AIH, diagnostic studies of the three autoantibodies might exhibit incorporation bias. Finally, we were unable to access valid original data to evaluate the diagnostic performances for AIH across the three indexes.

In conclusion, ANA provides moderate sensitivity and specificity, while SMA provides moderate sensitivity and high specificity, and anti-SLA/LP exhibits low sensitivity and high specificity. All three biomarkers remain limited by their unsatisfactory sensitivities and lack of consistency. Combining them may improve the diagnostic value, but laborious procedures would be required to establish the proper protocols. Without being able to explain the occurrence, expressions and pathogenesis of such disease, it will take a long time to identify an ideal indicator that would optimize sensitivity and minimize inconsistency in the diagnosis of AIH.

## Supporting Information

Checklist S1 **Preferred items reporting for the meta-analysis.**
(DOCX)
